# When Policy Meets Practice: Medical Residents and the Governance of Smartphone Use for Communication in Clinical Settings

**DOI:** 10.3390/healthcare14081061

**Published:** 2026-04-16

**Authors:** Neil G. Barr, Glen E. Randall

**Affiliations:** DeGroote School of Business, McMaster University, 1280 Main St. W., Hamilton, ON L8S 4M4, Canada; randalg@mcmaster.ca

**Keywords:** smartphones, physician communication, health policy

## Abstract

**Background/Objectives:** The use of personal smartphones by healthcare professionals in clinical settings has become a growing area of concern as practice may not consistently align with policy guidance. This study enhances our understanding of how and why medical residents are using smartphones to communicate patient healthcare information with other physicians in daily practice and provides insights into the role that institutional governance, policies, and structures play in the use of smartphones. **Methods:** This study used qualitative techniques to examine medical residents’ use of smartphones to communicate healthcare-related information with colleagues. Additionally, a neo-institutional theory lens was applied to assess the role that regulative (guidelines/policies), normative (what peers/staff are doing), and cultural-cognitive (beliefs/perceptions) factors play in smartphone use by medical residents. **Results:** The results suggest that behaviour related to smartphone use is based primarily on normative and cultural-cognitive factors rather than regulative factors. Regulative elements around smartphone use play a smaller role in shaping behaviour, particularly when they: (1) lack clarity; (2) are not seen as credible/legitimate; or (3) are viewed as cumbersome and do not align with workflow needs. **Conclusions:** The implementation of future guidelines/policies should consider the use of mentorships throughout postgraduate medical training whereby staff physicians educate, model, and promote behaviour in accordance with the associated policies/guidelines.

## 1. Introduction

The use of personal smartphones by healthcare professionals in clinical settings has become a growing concern, with calls for a more formalized governance of smartphones and related apps [1]. In addition to federal and provincial legislative requirements around privacy, the behaviours of healthcare professionals in Canada are largely governed by policies set by their respective provincial regulatory bodies, along with the policies of the various healthcare provider organizations where services are delivered. One area of potential conflict is the use of smartphones in clinical settings, where practice may not consistently align with policy guidance. Smartphones offer several advantages for communication among health care providers compared to traditional means, including timely access to information and potential increases in efficiency [2,3,4,5]. However, this technology can have disadvantages, such as disruptions to workflow and an increased risk of breaches in health information privacy [2,5,6,7]. Physicians are heavy users of smartphones for transmitting health information [8,9] and are therefore an important group to consider in assessing smartphone use and compliance with professional standards and organization policies.

To date, the study of smartphone use for communication of health information among physicians has been relatively narrow in scope. Most existing studies are survey-based and have failed to provide an in-depth understanding of how and why physicians use smartphones to exchange healthcare-related information with colleagues and their perceptions of the guidelines/policies governing smartphone use. Additionally, most studies do not incorporate an underlying theory of adopting smartphone technology, which limits the ability of researchers to explain outcomes and predict future effects. Research in this area would benefit from using qualitative approaches, such as interviews, and using theoretical perspectives to help us understand and shed light on these important factors. Existing theories in the social sciences have been used to explain individual differences in attitudes toward new technologies and practices (e.g., Theory of Planned Behavior) [10], and how innovations flow through various social networks (e.g., Diffusion of Innovations Theory) [11] or become embedded in everyday work (e.g., Normalization Process Theory) [12]. However, these do not consider the processes by which institutions become established as authoritative guidelines for social behaviour. Institutions are sets of rules, practices, and beliefs that shape the meaning and the perceived appropriateness of social behaviour. They organize states, societies, and international systems (e.g., governments, courts, constitutions) and, thus, are resistant to change. Along these lines, institutions “represent constraints on the options that individuals and collectives are likely to exercise, albeit constraints that are open to modification over time” [13] (p. 94).

Neo-institutional theory has been conceptualized in several ways across the social sciences (not limited to health care). In an attempt to bring some clarity to the heterogeneity that seems to dominate neo-institutionalist perspectives, Scott [14] distilled the various arguments down to a few central ideas. Initially, he proposed a broad definition of institutions so as to take into account the various arguments, and then sought to identify key elements that existed across the diverse perspectives. These key elements, called pillars, were based upon the most prevalent aspects of institutions, with the ultimate goal of constructing an analytic framework that facilitates examination of these factors in different contexts. This study applies his three-pillar framework to examine institutional influences on smartphone use in a health care context.

*The regulative pillar*. This element of institutions focuses on regularizing behaviour through rule-setting, monitoring, and sanctioning (e.g., force, coercion). Incentives can also be used to encourage compliance (e.g., through payments). Rules can be written/unwritten, and formal/informal (similar to etiquette in sport).

*The normative pillar*. This element is based on social obligations, with a focus on values (ideas regarding what is desirable/preferred) and norms (how things should be done). These constructs define the appropriate ways of obtaining desired goals (i.e., the rules of the game). Shared values and norms result in social order and the stability of that order, since institutions are seen as having moral roots [14].

*The cultural-cognitive pillar*. This element focuses on the interplay between cultural frames and individual cognition. In other words, to explain and understand behaviour, one needs to take into account how an individual interprets/perceives their conditions/environment.

Each of these pillars is useful in helping to explain social processes and behaviour by examining what individuals have to do (regulative), ought to do (normative), and want to do (cultural-cognitive). Neo-institutionalism suggests that social behaviour is controlled/guided not only by rules, but also by norms and cognitions. As Meyer and Rowan [15] argue, while formal structures (e.g., policies, rules) act as a “blueprint” for societal activity, neo-institutionalization involves the blending of rules, normative obligations, and social meaning. Although change may be brought about by developments outside of an organization (e.g., smartphones as a common way to communicate), these developments are ultimately filtered and shaped through an organization’s culture and the manner in which they are used by members of the organization [16]. Therefore, the relative influence of each of the three pillars of institutions is context-dependent.

Given postgraduate medical trainees’ (herein referred to as medical residents in the Canadian context) status within a hospital setting (e.g., frequently changing team/work environments, overload of information), there is reason to believe that the relative influence of the three pillars of institutions on smartphone use may be different for this group of health care professionals compared to others (i.e., regulative elements may play less of a role). Therefore, the application of a neo-institutional perspective could provide a greater understanding of the social processes driving the use of smartphones for communication among medical residents, which could help inform the development of future policies.

The purposes of this study were (1) to gain a greater understanding of how and why medical residents are using smartphones to communicate health care related information in daily practice; and (2) to provide insights regarding the role that institutional structures—regulative, normative, and cultural-cognitive—play in the use of smartphones for communication of health care related information by medical residents. This information will provide a better understanding of when and why there is a disconnect between policies and the behaviours of this group of frontline practitioners.

## 2. Materials and Methods

A case study was conducted with data being acquired through semi-structured interviews of medical residents. The need for case studies arises out of a desire to understand complex social phenomena within a dynamic and poorly understood setting. Yin [17] stresses the value of a case study approach when a phenomenon cannot be meaningfully understood outside of its real-world circumstances, which clearly applies to medical residents within a clinical environment. A case study approach allows investigators to gain an in-depth understanding of real-life events (e.g., small group behaviour) and to ask penetrating questions that seek to capture the richness of organizational behaviour [17]. This technique is recommended when an investigation: (1) seeks to answer “how” and “why” type questions; (2) does not attempt to control behavioural events; and (3) focuses on contemporary phenomena [17]. Likewise, individual semi-structured interviews were chosen as this technique “allows the interviewer to delve deeply into social and personal matters” [18] (p. 315). This approach is commonly used by researchers to obtain interviewee perceptions of experiences related to health care delivery [18].

Medical residents were selected due to their familiarity with smartphones and since they are more likely to use these devices in clinical practice than other groups of physicians (e.g., those who have been practicing for many years and, thus, have not used smartphones for most of their careers). Also, given their status within a hospital setting, the relative influence of institutional structures may be different for medical residents compared to other health care professionals. Therefore, medical residents represent a unique case and, thus, may reveal valuable insights about both unusual and typical behaviour. In addition, in the current body of literature that has examined smartphone use by physicians, most studies have focused on medical residents. Therefore, findings from this study will be comparable from a participant’s standpoint.

Purposeful sampling was used to select specific participants. The strength of this technique stems from studying information-rich cases in-depth, in which a researcher can learn “a great deal about issues of central importance to the purpose of the inquiry, thus the term purposeful sampling” [19] (p. 273). Medical residents from a single specialty training program in Ontario, Canada, were chosen for this study to minimize variations that may appear across specialties. The sample size/recruitment target for the study was based on the recommended number of participants needed to reach informational redundancy or theoretical saturation [20,21].

The study protocol, interview guide, and letter of information/consent form were approved by the Hamilton Integrated Research Ethics Board. Following research ethics approval, an invitation to participate in the study was sent via e-mail as per the postgraduate training program’s policy. The information in the e-mail provided a brief description of the study, including what study participation by the individual involved. If individuals were interested in participating or had any questions before making a decision about participating, they were asked to contact the Principal Investigator.

Prior to the interview, the PI reviewed the letter of information/consent form with each participant and gave them the opportunity to ask any questions or raise any concerns. The PI reaffirmed the participant’s willingness to be involved in the study and reminded them that they were free not to answer any question and could withdraw at any time. Subsequently, the participant and PI signed the consent form.

Interviews were 25–45 min in length and took place at a time and location convenient for the participant (two interviews were conducted over the phone, eight were conducted in a conference room). The interviews consisted of open-ended questions related to how and why smartphones are being used for communication of health care-related information among physicians, as well as awareness and knowledge of guidelines and policies governing the use of smartphones. Each interview was audio-recorded and transcribed. The transcripts were imported into NVivo 11 (software that supports the analysis of qualitative data).

Based on Roulston’s [22] description of the three phases of analyzing interview data—reduction, reorganization, and representation—as well as Schreier’s [23] suggestions for qualitative content analysis, the content of each interview was coded into themes by both authors with the aid of a coding guide. The initial guide was created prior to the analysis (based on the research/interview questions and anticipated themes), but was subject to modification (i.e., coding was an iterative process). In keeping with the iterative logic of qualitative content analysis, the authors repeatedly reviewed and discussed the coding guide and themes until agreement was reached.

## 3. Results

### 3.1. Participants

Ten medical residents from postgraduate years 2 to 4 of a single specialty training program in Ontario, Canada, took part in the study. Most participants stated that they worked approximately 60 h per week (with 50 being devoted to clinical activities and 10 to research, studying, and/or teaching). Most participants (9/10) used iPhones. One participant owned a Samsung smartphone. All were personal devices. None of the participants were sure if there was a policy stipulating that their smartphones be passcode-protected (or another form of “locking”), but they all used some sort of authorization protection (e.g., biometric recognition). In total, there were 12 major themes identified. Each is discussed below.

### 3.2. How Are Smartphones Being Used for Communication?

Participants reported using several applications/features of their smartphones for communication with other physicians. The most commonly used was text messaging (mainly via the messaging application WhatsApp), followed by e-mail, phone, and photos.

#### 3.2.1. Text Messaging

Text messages were used for relaying small pieces of information as quick updates to keep colleagues informed of various day-to-day activities, such as sharing clinically relevant lab results and ordering tests.

“So, texting I would use more for giving quick updates or checking in on where my team is, for example. So saying like, ‘I am going to go see a patient in bed three in acute.’ If there’s a lab value that comes back, you can say, ‘Potassium was 6.4,’ and then you can say, ‘Okay, let’s order this or this or this,’ or something like that. So it’s sort of quick blurbs.” (Participant 7)

Participants also described using text messaging because they believed it would be less disruptive to their colleagues than other forms of communication.

“Usually, texting is sort of the go-to because people are obviously in patient rooms, and you don’t want to be calling or paging and disturbing them, especially if it’s a non-urgent thing.” (Participant 10)

#### 3.2.2. E-Mail

Participants described using e-mail (primarily institutional accounts) mainly for administrative tasks, as opposed to communication about patient care (e.g., information about medical rounds, research, residency training program updates).

“[E-mail is] our primary contact for all sorts of information about our rotations and administrative things and evaluations… rotations and start times and scheduling and that sort of thing.” (Participant 5)

#### 3.2.3. Phone Calls

Participants discussed using the telephone feature of their smartphones for more urgent clinical issues or for exchanging relatively complex information that could not be conveyed by other means effectively.

“If I’m trying to give someone a more detailed context of a situation for a medical decision to be made, I probably will not use e-mail or text messaging. I’d probably call them and have that actual direct communication.” (Participant 3)

#### 3.2.4. Photos

Participants discussed taking photos on their smartphones as a way of sharing information—such as X-rays or ECGs—with other physicians (as it was perceived to be more convenient and timely to do so than by other means).

“Sometimes we get an admission who is transferred and their X-ray is not necessarily on our system, but we have the disc, so we’ll take a picture of the X-ray, and send it to our staff so that they can see an image in a timely manner.” (Participant 7)

In addition, pictures were taken with smartphones to document—or to track the progress of—interesting clinical findings for teaching purposes.

“Photos I’ve used occasionally for things like teaching purposes, like if it’s a patient with an interesting sort of physical finding, we can take a photo, but for that we require permission from the parents/patient and so we would get that before taking a photo.” (Participant 5)

### 3.3. Why Are Smartphones Being Used for Communication?

Participants described several reasons for using their smartphones instead of other communication methods. Based upon the analysis of interview data, two main themes emerged: (1) efficiency and (2) convenience.

#### 3.3.1. Efficiency

Many participants stated that smartphone use saved time and allowed for faster communication (compared to other methods of communication).

“So I find that it’s more efficient, and it’s just easier to use, particularly for those short questions because, really, the other alternative is paging someone and waiting for them to page you back. And that can sometimes turn into a little bit of phone tag, whereas if it’s a sort of short, kind of quick, thing, it’s way more efficient to text them and say, ‘Hey, this is what happened’.” (Participant 8)

#### 3.3.2. Convenience

Participants also alluded to the fact that smartphones were easier to use than other methods of communication.

“The amount of time that I’m in a single place on any given day is very small… It’s much easier to carry this [a smartphone] around than my laptop. And it’s just quicker than any of the institutional computers. I can’t emphasize enough how slow it is to log into an institutional computer… particularly on busy services, it’s just not feasible to do that between every single patient.” (Participant 9)

Greater efficiency and convenience in communication (through the use of smartphones) were perceived by participants to be associated with benefits related to the facilitation of patient care. These perceived benefits included improvements in communication, team function, and quality of care.

#### 3.3.3. Improved Communication

Several participants noted that smartphone use may help facilitate better communication.

“I think that the number one reason that mistakes get made in medicine is communication errors. So, having a good tool for communication is really helpful. And I think nothing replaces face-to-face communication, but I think especially when you’re sort of on a team of people and trying to interact, it really does help to have that sort of instant feedback and instant communication; smartphones allow for that.” (Participant 5)

#### 3.3.4. Improved Team Function

Given the volume of information that is exchanged among team members, interviewees felt that smartphones facilitated teamwork by keeping colleagues up-to-date with various patient care activities.

“We all work in a team-based format, so a lot of the time, it’s just checking in to make sure that everyone is either doing their job or what has been done so that the team can actually function as a team.” (Participant 3)

#### 3.3.5. Improved Quality of Care

Some participants felt that using smartphones enhanced the quality of care that they provided.

“So the expectation of patients now, or for as long as I’ve been practicing, which is not very long—maybe they’ve always had this expectation—is that they get timely, appropriate, informed care. Smartphone use helps us meet that expectation.” (Participant 9)

### 3.4. The Three Pillars and Smartphone Use (A Neo-Institutional Perspective)

As an initial step toward gaining insight into the role that regulative, normative, and cultural-cognitive institutional elements play in the use of smartphones for communication of health care-related information by medical residents, participants were asked to recall any guidance they received regarding the use of their smartphones. Most participants stated that training in this capacity was mainly informal, such as conversations among colleagues.

“I don’t know if I’ve ever had formal training. But just from being around other physicians, just general professionalism with cell phones of not using it when you’re having a discussion with other physicians or with other colleagues or definitely with patients. And also the privacy issues that come with cell phones. So we’ve had lots of talk about that… it’s more sort of you see what others are doing, and it’s more verbal and they sort of tell you whether it’s appropriate or not.” (Participant 1)

When delving deeper into the possible reasons why medical residents were not aware of the specific guidelines available regarding smartphone use (i.e., the extent or nature of direction provided), several participants noted that the number of guidelines they are expected to know is so large that, inevitably, some will receive more attention than others.

“I think for me, personally, it’s just the volume of information we’re inundated with, and that there’s a guideline for everything under the sun, and there’s a regulation for everything under the sun. And it’s just so hard to keep on top of it. And as a resident, not only are you trying not to break any rules, you’re also trying not to kill anyone, and trying to maintain some sort of sense of sanity in your work and in your life. And the volume of it’s so high that things get forgotten and things get missed.” (Participant 10)

Given that medical residents may not be well versed in the specific guidelines governing smartphone use, how do they determine appropriate ways of using their smartphones? Throughout the interviews, it became evident that smartphone use was based primarily on normative elements (professional norms; behaviour of peers/staff) and/or cultural-cognitive factors (medical residents’ beliefs/perceptions regarding facilitation of task completion and, hence, patient care), while regulative elements (guidelines/policies) were less impactful.

Based on further examination of these findings through an analysis of interview data, three themes emerged that help to explain some of the circumstances under which regulative elements may play a smaller role in shaping behaviour. These are: (1) when there is a perceived lack of clarity in rules; (2) when guidelines/policies are not seen as credible/legitimate; and (3) when guidelines/policies are viewed as cumbersome and do not align with workflow needs. Each of these themes will be discussed in turn.

#### 3.4.1. When There Is a Perceived Lack of Clarity in Rules

When there is uncertainty and/or ambiguity in guidance for social behaviour (i.e., smartphone use), medical residents appear to rely on senior staff to know what is best (i.e., the most appropriate way of behaving).

“So first of all, I base a lot on being a resident and a trainee. I base a lot of what I do on what my staff’s comfortable with, because I think there’s a lot of ambiguity in all of this. And nobody’s really sure what they can do. Nobody’s really sure if they can send patient initials or room numbers. And so because of that, different staff have different preferences, so a lot of what I do is because my staff want me to.” (Participant 2)

#### 3.4.2. When Guidelines and Policies Are Not Seen as Credible/Legitimate

Many participants expressed the view that some of the guidelines for smartphone use seemed illogical, as they did not take into consideration the applied or real-world (clinical) applications of the technology. This appeared to lead to the perception that such guidance was not convincing or justified.

“I think if you want people to be aware of guidelines and use those guidelines, you have to harmonize the guidelines with the reality of what’s happening. And having an administrator that I’ve never met, who feels very far away from the day-to-day life of being in the hospital, come down from Internet heaven and tell us how to use our smartphones is, I think, a little bit… I don’t think you’ll have a lot of success with that method. Like telling a group of residents, 97% of whom use iPhones, to all start using BlackBerry Messenger. That sort of thing, I think, really undermines the credibility of those guidelines.” (Participant 9)

This perceived lack of credible or legitimate guidelines appeared to cause a lack of trust in those who developed the guidelines. In these situations, medical residents look to what the senior staff are doing (norms), as they feel they can trust them:

“But that’s what I mean: with the guidelines, that doesn’t seem justified. The same people who make clearly insane decisions like that, I’m not necessarily interested in what they say about how I should use my smartphone when the people that I work with, who are senior to me, whom I trust, use their smartphones in this way.” (Participant 6)

Other participants illustrated the idea of already knowing how to behave appropriately by comparing the difference between clinical guidelines and guidelines for smartphone use.

“There are many clinical guidelines that people ignore, and people ignore clinical guidelines when they feel like they know what the right thing to do is. I think physicians ignore smartphone guidelines when they don’t align with what physicians need to do to get through the day. So I go to a guideline, a clinical practice guideline, when there’s no good evidence to tell me what the right thing to do is. I think a lot of people feel like they have very good common-sense evidence about how to use their smartphone.” (Participant 9)

This finding is in keeping with Scott’s [14] claim that to be seen as legitimate, institutions—or institutional carriers such as standards and guidelines—need social acceptability and credibility.

#### 3.4.3. When Guidelines/Policies Are Viewed as Cumbersome and Do Not Align with Workflow Needs

Interviewees also indicated that using the existing information and communication technology (ICT) resources within the hospital inhibited efficient completion of various tasks.

“So an ECG gets done on a machine that is DOS-based from 1979 or something like that. … I suppose I could scan the ECG to my (hospital) e-mail and then forward it from my (hospital) e-mail to somebody else’s e-mail, but again, that involves realistically another 20 min of time… everyone works around the processing like crazy, but nobody acknowledges how insane that is.” (Participant 9)

Given the growing complexity and sophistication of ICT, it appears as though some components of hospital ICT infrastructure are unable to match the “capability” of smartphones. As previous research has indicated, current university and hospital policies governing the use of digital devices may be outdated, confusing/vague, and do not align with day-to-day patient care activities [24]. Therefore, to meet the expectations of a complex health system, medical residents implement “workarounds” to traditional (hospital-based) communication methods (i.e., the use of personal mobile computing, which has been shown to increase workflow efficiency) [25].

## 4. Discussion

Scott’s institutional framework provides a useful foundation for understanding how organizations respond to external pressures through regulative, normative, and cultural-cognitive mechanisms. However, less attention has been paid to how institutional pressures are interpreted by middle managers, or in this case, medical residents, within a complex system such as healthcare. The responses from medical residents provided several insights regarding their use of smartphones for health care-related communication among colleagues, including how they are used, the underlying reasons for use, and the relative influence of regulative, normative, and cultural-cognitive institutional elements on use. Interview data suggested that medical residents are aware that, in addition to the federal and provincial legislative requirements related to privacy, there are guidelines and policies that govern the use of smartphones for work-related activities. However, when it comes to formalized documents such as legislation or organizational policies, they are unsure of the specific direction that is provided. Given their focus on efficiency and convenience, coupled with guidelines/policies for smartphone use that are perceived as not providing clear direction for appropriate behaviour or are not seen as credible/legitimate, this study suggests that medical residents model their behaviour primarily based on what colleagues are doing, or act in a manner that they believe facilitates task completion and, hence, patient care. Regarding the latter, existing alternatives to smartphone use for communication are less appealing (the hospital infrastructure/resources conflicts with day-to-day practice/workflow). In this way, the normative (behaviour of colleagues/staff; what an individual ought to do) and cultural-cognitive (medical residents’ beliefs/perceptions; what an individual wants to do) elements of institutions appear to influence the use of smartphones by medical residents more so than regulative elements (guidelines/policies; what an individual has to do) of institutions.

From a practical standpoint, given the apparent disconnect between guidelines/policies regarding the use of smartphones and the use/need of smartphones by medical residents, there is a need to examine the prospect (and outcomes) of shared decision-making among policymakers and health care providers. Furthermore, as the use of ICT challenges existing work processes and social interactions among providers and patients, further research focusing on the interactive relationships among patients, professionals, and organizational systems is needed [26]. This avenue would be in keeping with suggestions from other authors that institutional researchers should explore how micro-level organizational cultural dynamics influence, and are influenced by, the macro-level institutional context [27]. It would also be fruitful to examine the perspectives of patients and health care managers to gain an understanding of how the use of smartphones by medical residents is viewed; in the case of patients, whether they are actually concerned with their health information being sent through potentially unsecure smartphone channels, and if the etiquette used by medical residents is acceptable; in the case of managers, what the unique challenges are in governing the use of smartphones.

From a theoretical standpoint, a considerable impediment to the use of an institutional perspective is that the term has been interpreted in numerous ways [28]. The application of a comprehensive interpretation of institutions, such as the one used in this study, will help strengthen the contribution of this perspective when explaining social phenomena. Such an expansive viewpoint, particularly in the ICT domain, has been recommended for some time. As King asserted, “long-established intellectual perspectives on innovation from neoclassical economics and organization theory are inadequate to explain the dynamics of actual innovative change in the IT (information technology) domain. A broader view adopted from economic history and the new institutionalism in sociology provides a stronger base for understanding the role of institutions in IT innovation” [29] (p. 139).

The findings from this study suggest that medical residents perceive guidelines for smartphone use as not taking into account the day-to-day clinical and practical applications that these devices provide. As a result, there is a mistrust of associated policies. This circumstance may create resistance from medical residents and, correspondingly, a negative perception of “rules” related to the use of smartphones. Therefore, to ensure the successful integration of ICT, such as smartphones, the characteristics of providers and of the local context need to be taken into account. Organizations start by adopting required ICT changes and then begin internalizing such changes, which leads to an incremental impact of additional institutional influences occurring [30]. Therefore, there is a need to routinely take stock of and account for the day-to-day use of new technology by providers. By doing so, guidelines can be developed that “resonate” with the associated clinical uses of the technology, fostering their uptake.

As medical residents frequent multiple teams and locations in the hospital as part of their training, in a sense, they lack the structure of other health care providers; the workplace climate changes relatively frequently, which requires a certain amount of adaptation on their part. In the absence of such consistency, there may be a greater likelihood of basing one’s behaviour on what others are doing in similar circumstances, or what one believes to be the most appropriate behaviour (i.e., the logic of appropriateness that individuals acquire through their membership in institutions) [14]. Consequently, the implementation of future policies could consider continuing education via physician mentors (or a champion within the medical staff) to model and promote behaviour in accordance with the associated guidelines.

Some researchers have recommended that greater emphasis be placed on digital professionalism in medical education (i.e., there is a need for a comprehensive model of proper use of ICT in a health care context). By ignoring the challenges that medical trainees face when using ICT, there is a risk of perpetuating “a hidden curriculum of digital media use, framing the use of digital media in terms of problems and risks while ignoring the ways in which it can support and shape medical practice” [31] (p. 844). Health care managers and senior staff (physician mentors) could play a central role in promoting digital professionalism, preparing medical residents for the anticipated cultural changes, and lending support to them as they proceed through their training. In this vein, organizations should employ strategies to help individuals working within them to use technologies in the most appropriate way [7,9,32] and could look to other organizations/hospitals or industries for insight.

### Limitations

While purposeful sampling was used to study an information-rich case in-depth, medical residents from only one specialty at one institution were enrolled in the study. As a result, a broader array of experiences and opinions was not captured. The inclusion of other medical specialties at other institutions may have provided additional insights (as alluded to earlier in this paper, the influence of institutional elements is context dependent). Additionally, member-checking was not employed. This process involves bringing the data back to the participants with the identified themes and asking them if they would like to provide further explanations or greater detail. Such a procedure ensures that what the researcher has developed fits with the experiences of the participants. Given the busy schedules of medical residents, member-checking was not conducted to avoid overburdening the participants (i.e., it was viewed as not being feasible).

## 5. Conclusions

As the use of smartphones in clinical settings expands, so do the concerns related to their use and the volume of corresponding research around how and why they are being used [33]. In the current study, it is clear that medical residents rely heavily on the use of personal smartphones to communicate healthcare-related information with colleagues as they perceive these devices to be more efficient and convenient than traditional means. By applying a neo-institutional perspective, this study has provided a greater understanding of the relative influence of regulative, normative, and cultural-cognitive institutional elements on smartphone use by medical residents. More specifically, medical residents base their smartphone use behaviour primarily on normative elements (professional norms; what peers/staff are doing) and cultural-cognitive elements (beliefs/perceptions regarding facilitation of task completion). Regulative elements (guidelines/policies) around smartphone use play a smaller role in shaping behaviour, particularly when they: (1) lack clarity; (2) are not seen as credible/legitimate; or (3) are viewed as cumbersome and do not align with workflow needs. In this vein, findings from this investigation indicate that there is a disconnect between perceived constraints of policies/guidelines for smartphone use and the realities of daily life for busy junior physicians in complex health care teams. Consequently, there is a need to promote knowledge exchange between “developers/providers” and “consumers/users” of guidelines/policies pertaining to the use of smartphones by medical residents.

However, improving guidelines/policies, while necessary, is likely insufficient given that professional norms and medical residents’ beliefs/perceptions are important influences on their behaviour. Therefore, the implementation of future guidelines/policies should consider the use of mentorships throughout postgraduate medical training whereby staff physicians (or a champion within the medical staff) educate, model, and promote behaviour in accordance with the associated guidelines. Future research should examine the outcomes of such efforts and explore strategies for standardizing these initiatives across organizations.

As efforts to improve health systems continue, the role that smartphones play cannot be underestimated. This claim is exemplified in the following words of one study participant: “If you took away all the smartphones from the residents at least in the hospital, the hospital would grind to a halt.” (Participant 9) This is but one indication that the smartphone may one day be recognized as a tool that is “as irreplaceable as the stethoscope has been in the practice of medicine” [34] (p. 11).

## Data Availability

The data cannot be made publicly available due to privacy concerns, as the interview materials contain identifying information. However, the data presented in this study are available from the corresponding author upon reasonable request.

## Abbreviations

The following abbreviations are used in this manuscript:

PI	Principal investigator
ICT	Information and communication technology
